# Invasive pneumococcal disease among adults in Japan, April 2013 to March 2015: disease characteristics and serotype distribution

**DOI:** 10.1186/s12879-016-2113-y

**Published:** 2017-01-03

**Authors:** Munehisa Fukusumi, Bin Chang, Yoshinari Tanabe, Kengo Oshima, Takaya Maruyama, Hiroshi Watanabe, Koji Kuronuma, Kei Kasahara, Hiroaki Takeda, Junichiro Nishi, Jiro Fujita, Tetsuya Kubota, Tomimasa Sunagawa, Tamano Matsui, Kazunori Oishi, the Adult IPD Study Group

**Affiliations:** 1 Field Epidemiology Training Program, National Institute of Infectious Diseases, Tokyo, Japan; 2 Department of Epidemiology for Infectious Diseases, Osaka University Graduate School of Medicine, Osaka, Japan; 3 Department of Bacteriology I, National Institute of Infectious Diseases, Tokyo, Japan; 4 Division of Infection Control and Prevention, Niigata University Graduate School of Medical and Dental Sciences, Niigata, Japan; 5 Department of Infection Control and Laboratory Diagnostics, Internal Medicine, Tohoku University Graduate School of Medicine, Miyagi, Japan; 6 Department of Medicine, National Hospital Organization, Mie Hospital, Mie, Japan; 7 Department of Infection Control and Prevention, Kurume University School of Medicine, Fukuoka, Japan; 8 Department of Respiratory Medicine and Allergology, Sapporo Medical University School of Medicine, Hokkaido, Japan; 9 Center for Infectious Diseases, Nara Medical University, Nara, Japan; 10 Department of Respiratory Medicine, Yamagata Saisei Hospital, Yamagata, Japan; 11 Department of Microbiology, Kagoshima University Graduate School of Medical and Dental Sciences, Kagoshima, Japan; 12 Department of Infectious Diseases, Respiratory and Digestive Medicine, Faculty of Medicine, University of the Ryukyus, Okinawa, Japan; 13 Department of Hematology and Respiratory Medicine, Kochi Medical School, Kochi University, Kochi, Japan; 14 Infectious Diseases Surveillance Center, National Institute of Infectious Diseases, Tokyo, Japan

**Keywords:** *Streptococcus pneumoniae*, Invasive pneumococcal disease, Indirect effect, Surveillance

## Abstract

**Background:**

In Japan, the clinical characteristics and recent serotype distribution among adult patients of invasive pneumococcal disease (IPD) have not been fully investigated since the introduction of the pneumococcal conjugate vaccine (PCV) in children. From November 2010, PCV7 was encouraged by an official program, funded by government, subsequently included in the routine schedule in April 2013, and replaced with a PCV13 in November 2013.

**Methods:**

Between April 2013 and March 2015, patients with IPD older than 15 years were evaluated based on the enhanced national surveillance in ten prefectures of Japan. The serotype distribution of the isolates was analyzed in these patients.

**Results:**

The analysis included 291 patients: 107 patients (37%) were female and the median age was 70 years. Of 281 patients with available data, 202 (72%) had underlying diseases, including 107 patients (38%) with immunocompromised status. The case fatality proportion for all case was 20%. In subgroup analysis, the case fatality proportion (29%) in immunocompromised patients was much higher than that (0–16%) in each age group of nonimmunocompromised patients (15–39 years, 40–64 years, and ≥ 65 years). While the proportion of bacteremia without any focus (27%) was higher than that (8–10%) in nonimmunocompromised patients, the proportions of vaccine types (PCV13, 32%; PPSV23, 51%) of the causative isolates were lower than those in each age group of nonimmunocompromised patients. Among 291 isolates, the most frequent serotypes were 3 (17%), 19A (13%), and 22F (10%). Twelve percent of the isolates were PCV7 serotypes, 46% were PCV13 serotypes, and 66% were PPSV23 serotypes.

**Conclusions:**

The majority of adult patients of IPD had underlying diseases, including immunocompromised conditions. A low proportion (12%) of PCV7-type IPD was observed in this population where PCV7 for children had been included in the routine immunization schedule.

**Electronic supplementary material:**

The online version of this article (doi:10.1186/s12879-016-2113-y) contains supplementary material, which is available to authorized users.

## Background

*Streptococcus pneumoniae* frequently colonizes the nasopharyngeal tract of children and causes noninvasive infections, such as otitis media and nonbacteremic pneumonia. Furthermore, it can cause meningitis, bacteremic pneumonia, bacteremia/septicemia, and other invasive pneumococcal diseases (IPDs) [1, 2].

The introduction of a heptavalent pneumococcal conjugate vaccine (PCV7) has dramatically reduced the incidence of invasive pneumococcal disease (IPD) among vaccinated young children [3–6] and, as a result of herd immunity, has decreased IPD among the elderly. However, non-PCV7-type IPD has increased 35 years after PCV7 introduction among adults. This increase may have occurred because of serotype replacement, a phenomenon in which the prevalence of nonvaccine serotypes rises while that of vaccine serotypes falls [7].

PCV7 was approved for voluntary vaccination for children in February 2010 in Japan. From November 2010, PCV7 vaccination was further encouraged for children aged <5 years by an official program, the Provisional Special Fund for the Urgent Promotion of Vaccination by the Japanese government. PCV7 was included in the routine schedule in April 2013, and replaced with a 13-valent pneumococcal conjugate vaccine (PCV13) in November 2013. Consequently, a high vaccination rate (94.2%) was observed in children at 24 months of age in 2015 [Sakiyama H, Oishi K, unpublished data]. For adults, a 23-valent pneumococcal polysaccharide vaccine (PPSV23) was approved in 1988 and included in routine immunization in October 2014 for individuals aged 65 years or older, and PCV13 was approved for adults aged 65 years or older in June 2014, on a voluntary basis. After the introduction of PCV7, a decrease of 98% in the incidence of IPD caused by the PCV7 serotypes was reported in Japan [8]. This resulted in the 57% decline in overall IPD, although an increase of IPD incidence caused by PCV13 minus PCV7 serotypes and non-PCV13 serotypes among children younger than 5 years of age was observed [8]. However, the epidemiology of IPD in Japan, including its serotype distribution among adults, after the introduction of PCV for children remains unknown.

In this paper, we describe the disease characteristics and serotype distribution of pneumococcal isolates in IPD among adults in Japan in the period of April 2013-March 2015.

## Methods

### Study design

A national surveillance program (National Epidemiological Surveillance of Infectious Diseases: NESID) for IPD has been in place under the Infectious Disease Control Law of Japan since April 2013. Physicians in all clinics and hospitals are required to notify all cases to local public health authorities within 7 days of diagnosis. A case of IPD is defined as detection of *S. pneumoniae* by bacterial culture, *S. pneuemoniae*-specific DNA by PCR in blood and/or cerebrospinal fluid (CSF) or a positive reaction of latex agglutination test or immunochromatography test in CSF. Data fields collected from cases include demographic information, syndrome, exposure history, history of pneumococcal vaccination and evidence for diagnosis, and were recorded in the NESID system. The Adult IPD Study Group (http://www.nih.go.jp/niid/ja/ibi.html) conducted enhanced surveillance of adult IPD based on the NESID in ten of 47 prefectures in Japan, to assess the clinical characteristics and serotype distribution of pneumococcal isolates between April 2013 and March 2015. The standardized case-reporting form for this enhanced surveillance included sociodemographic and clinical data on age, sex, body mass index (BMI), smoking history, alcohol abuse, history of PPSV23 vaccination, preceding infection of influenza, current underlying diseases, immunocompromised conditions, and outcome.

We enrolled all adult IPD patients (defined as IPD patients older than 15 years of age) from the ten enhanced surveillance prefectures (Hokkaido, Miyagi, Yamagata, Niigata, Mie, Nara, Kochi, Fukuoka, Kagoshima, and Okinawa prefectures) in Japan. The ten prefectures were chosen for their relatively even distribution across the country and willingness to participate in the study (Additional file 1). The IPD patients older than 15 years of age reported to NESID from the ten enhanced surveillance prefectures comprised 21% (567/2752; unpublished data) of the total reported IPD patients older than 15 years of age in Japan over the study period. In study sites, a physician who diagnosed a patient as having IPD notified the local public health department and enhanced surveillance personnel in the home prefecture of the patient. The case fatality was defined as reporting of death by the case-reporting form at the time of notification in this study. These case-reporting forms were collected from physicians, then sent to the Infectious Diseases Surveillance Center (IDSC) at the National Institute of Infectious Diseases (NIID) for analysis. One isolate per patient was included in this analysis.

### Serotyping

Isolated strains from blood and/or CSF were sent to the prefectural and municipal public health institutes from hospitals located in the study area, in cooperation with local public health centers. Some pneumococcal isolates were serotyped by multiplex serotyping PCR at the prefectural public health institutes [9]. All pneumococcal isolates, including isolates serotyped by multiplex serotyping PCR at the prefectural public health institutes, were sent to the Department of Bacteriology I, NIID and serotyped by capsule quelling reaction with rabbit antisera (Statens Serum Institute, Copenhagen, Denmark), as described elsewhere [10].

### Descriptive analysis

Data were entered, managed, and analyzed using MS Excel. We calculated the proportions of recorded IPD patient characteristics stratified by age and immunocompromised status in four groups: group 1: 15–39 years, non-immunocompromised; group 2: 40–64 years, nonimmunocompromised; group 3: ≥ 65 years, nonimmunocompromised; and group 4: ≥ 15 years, immunocompromised.

## Results

### Study population and clinical features of IPD patients

Between April 2013 and March 2015, a total of 303 IPD cases (101 cases from April 2013 to March 2014, 202 cases from April 2014 to March 2015) were registered in the ten enhanced surveillance prefectures, among cases reported to NESID from the ten prefectures (*n* = 567; unpublished data) in study period. Among the 303 reported IPD cases, bacterial culture of blood or CSF was positive for *S. pneumoniae* in 301 cases at each medical institution. In two cases, PCR test targeting *lytA* was positive (blood or CSF sample was positive for each one case) at NIID [9]. No discordant results were found in 124 isolates between the isolates serotyped by capsule quelling reaction with rabbit antisera at NIID and those serotyped by multiplex serotyping PCR at the prefectural public health institutes. Among the 301 cases with positive bacterial culture, pneumococcal isolates were not collected at NIID in six cases and the case-report form was not obtained in four cases. We therefore analyzed 291 cases (96%) for whom both case-report data and isolate data were available.

The characteristics of the 291 IPD patients are summarized in Table 1; 37% were female, the median age (interquartile range, IQR) was 70 (61–81) years, and 65% of patients were aged ≥65 years. The proportion of patients vaccinated with PPSV23 within the previous 5 years was 7%. No patients had a vaccination history of PCV13. Comorbidity was reported in 72% of the 281 patients, and 38% had at least one immunocompromised status, of which malignancy (20%) was the most common. Among 173 pneumonia cases, 170 cases (98%) were diagnosed based on pulmonary infiltration in chest radiograph and/or chest computed tomography and three cases were diagnosed by isolation of *S. pneumoniae* on sputum sample. Among 46 meningitis cases, 83% were diagnosed by isolation of *S. pneumoniae* or detection of pneumococcal antigen from CSF; eight cases were diagnosed by blood culture and the presence of meningeal signs. The case fatality proportion for all cases was 20% at the time of reporting. The median time from patient onset to case reporting was 35 (IQR, 20–94) days. Among fatal cases, 77% (44/57) had underlying diseases.

**Table 1:** Characteristics of IPD patients in 10 prefectures in Japan (*n* = 291) from April 2013 to March 2015

Variable	n/N^a^ (%)
Female	107/291 (37)
Median age; years (IQR)	70 (61-81)
Age group	
15–39 years	15/291 (5)
40–64 years	86/291 (30)
≧65 years	190 (65)
BMI	
Normal or Healthy Weight (18.5 – 24.9)	139/248 (56)
Overweight (25.0 and Above)	41/248 (17)
Underweight (Below 18.5)	68/248 (27)
Smoking history	132/256 (52)
Alcohol abuse	39/257 (15)
Preceding influenza	11/192 (6)
Vaccination history of PPSV23	16/231 (7)
Comorbidities	202/281 (72)
Chronic pulmonary disease	24/281 (9)
Chronic heart disease	19/281 (7)
Chronic liver disease	19/281 (7)
Chronic kidney disease	17/281 (6)
Diabetes mellitus	34/281 (12)
Immunocompromised status	107/281 (38)
Hyposplenia/asplenia	14/271 (5)
Autoimmune disease	33/281 (12)
Corticosteroid therapy	26/281 (9)
Malignancy	56/281 (20)
Solid cancer	37/281 (13)
Hematologic cancer	17/281 (6)
Anti-cancer agent	22/281 (8)
Clinical presentation	
Bacteremia without any focus	47/291 (16)
Meningitis	46/291 (16)
Bacteremic pneumonia	173/291 (59)
Others^b^	25/291 (9)
Serotype	
PCV13 type	135/291 (46)
PPSV23 type	191/291 (66)
Outcome^c^	
Fatal	57/291 (20)

*IQR* Interquartile range, *BMI* Body mass index, *PPSV23* 23-valent pneumococcal polysaccharide vaccine

^a^ Number of available answers

^b^ Others included bacteremic arthritis, bacteremic cellulitis, bacteremic spondylitis bacteremic cholangitis, bacteremic empyema, infected aortic aneurysm and infective endocarditis

^c^ The case fatality was defined as reporting of death by the case-reporting form at the time of reporting

### Clinical features of IPD patients stratified by age and immunocompromised status

The characteristics of the 281 IPD patients stratified by age and immunocompromised status are shown in Table 2. The proportion of bacteremia without any focus (27%) was higher in Group 4 (immunocompromised patients) than that in Group 1, 2 or 3 (each group of nonimmunocompromised patients). The case fatality proportion in Group 4 (29%) was much higher that (0–16%) in Group 1, 2 or 3. The proportions of vaccine types (PCV13, 32%; PPSV23, 51%) of the causative isolates in Group 4 were lower than those in Group 1, 2 or 3.

**Table 2:** Characteristics of IPD patients by age and immunocompromised status (*n* = 281)

	Group 1 *n* = 10 (15–39 years, Nonimmunocompromised)	Group 2 *n* = 48 (40–64 years Nonimmunocompromised)	Group 3 *n* = 116 (> = 65 years, Nonimmunocompromised)	Group 4 *n* = 107 (> = 15 years Immunocompromised)
Variable	n/N^a^ (%)	n/N^a^ (%)	n/N^a^ (%)	n/N^a^ (%)
Female	3/10 (30)	10/48 (21)	46/116 (40)	44/107 (41)
BMI				
Normal or Healthy Weight (18.5 – 24.9)	5/7 (71)	24/42 (57)	57/95 (60)	47/97 (48)
Overweight (25.0 and Above)	0/7 (0)	11/42 (26)	12/95 (13)	17/97 (18)
Underweight (Below 18.5)	2/7 (29)	7/42 (17)	26/95 (27)	33/97 (34)
Smoking history	5/9 (56)	26/44 (59)	49/100 (49)	48/95 (51)
Alcohol abuse	2/9 (22)	13/43 (30)	13/103 (13)	11/95 (12)
Preceding influenza	0/4 (0)	2/34 (6)	8/83 (10)	1/66 (2)
Vaccination history of PPSV23	0/8 (0)	1/40 (3)	6/91 (7)	9/85 (11)
Comorbidities	1/10 (10)	25/48 (52)	69/116 (59)	107/107 (100)
Clinical presentation				
Bacteremia without any focus	1/10 (10)	4/48 (8)	12/116 (10)	29/107 (27)
Meningitis	3/10 (30)	9/48 (19)	18/116 (16)	14/107 (13)
Bacteremic pneumonia	6/10 (60)	27/48 (56)	80/116 (69)	54/107 (50)
Others^b^	0/10 (10)	8/48 (17)	6/116 (5)	10/107 (9)
Outcome^c^				
Fatal	0/10 (0)	6/48 (13)	18/116 (16)	31/107 (29)
Serotype				
PCV13 type	4/10 (40)	27/48 (56)	65/116 (56)	34/107 (32)
PPSV23 type	7/10 (70)	38/48 (79)	85/116 (73)	55/107 (51)

*BMI* Body mass index, *PPSV23* 23-valent pneumococcal polysaccharide vaccine

^a^ Number of available answers

^b^ Others included bacteremic arthritis, bacteremic cellulitis, bacteremic spondylitis bacteremic cholangitis, bacteremic empyema, infected aortic aneurysm and infective endocarditis

^c^ The case fatality was defined as reporting of death by the case-reporting form at the time of reporting

### Serotype distribution of pneumococcal isolates

**Fig. 1:**
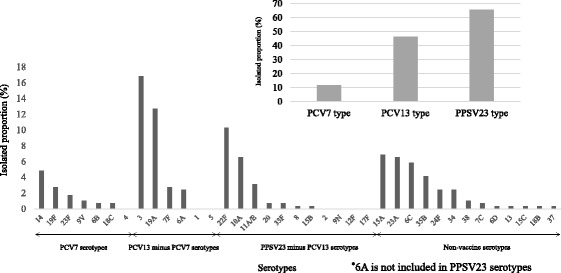
Distribution of the serotypes of causative pneumococcal isolates (*n* = 291) from patients with invasive pneumococcal diseases from 10 prefectures in Japan, from April 2013 to March 2015 grouped by vaccine serotype

Among the 291 isolates, the most frequent causative serotype was 3 (49 isolates; 17%), followed by 19A (37 isolates; 13%) and 22F (30 isolates; 10%). No nontypeable pneumococcal isolates was found. Twelve percent of the isolates were PCV7 serotypes, 46% were PCV13 serotypes, and 66% were PPSV23 serotypes (Fig. 1). The PPSV23 minus PCV13 serotypes accounted for 22%. Non-vaccine serotypes 15A (20 isolates; 7%), 23A (19 isolates; 7%), 6C (17 isolates; 6%), and 35B (12 isolates; 4%) were also frequently isolated. The proportions of non-vaccine types of the causative organisms in Group 4 were higher that in Group 1, 2 or 3 (Additional file 2). Among the 16 IPD patients who had received a vaccination with PPSV23 within 5 years of the onset of illness, six (38%) had disease caused by PPSV23 serotypes, such as 3, 14, 7F, 22F, 23F, and 33F.

## Discussion

This study reports the characteristics and serotype distribution of causative isolates among adult patients with IPD. The majority of patients had underlying diseases, including immunocompromised conditions.

The proportion of patients in our study whose IPD was preceded by influenza (6%) was in agreement with a previous study reported in the US [11]. As the current proportion of vaccination with PPSV23 among the elderly (older than 65 years of age) in Japan is estimated to be approximately 33% (estimated by the reported number of shipped doses of PPSV23 from MSD K.K. Japan between Jan 1st 2009 and March. 31th 2015/Japanese population older than 65 year-old (Oct. 1st 2012 national statistics [12], the proportion of vaccination with PPSV23 (9%; 13/150) in adult IPD patients ≥ 65 years of age found in this study was very low. This low vaccination proportion is likely explained by the reason why we calculated the vaccination proportion based on IPD case series; however, these results indicate that it is critical to encourage the unvaccinated elderly to receive a PPSV23 vaccination via the routine immunization program.

The results of our study suggest that the introduction of PCV7 followed by PCV13 in children may have reduced the coverage proportions of PCV13 and PPSV23 from 61% and 85% to 46% and 66%, respectively, compared with the proportions observed in 2006–2007 [13]. Although the surveillance sites and the method of sample collection differed between the two studies, the factors that affect serotype distribution, such as age and comorbidities, were found to be equivalent. From November 2010, PCV7 was promoted by the Japanese government for children aged < 5 years by the Provisional Special Fund for the Urgent Promotion of Vaccination. Thereby, all children aged < 5 years were subsidized for the vaccination of PCV7. Since then, vaccination coverage of PCV7 has increased among children in Japan. Subsequently, serotype replacement in children after PCV7 introduction was reported from the prospective population based on surveillance from 2008 to 2013 among 10 selected prefectures in Japan [8], of which seven prefectures were included in our study sites. Furthermore, a recent publication reported that the coverage proportions of PCV13 and PPSV23 had decreased for isolates from adult cases of IPD between 2010 and 2013, as assessed by hospital-based surveillance performed in Japan [14]. Our study of adult IPD, which was performed between April 2013 and March 2015, demonstrated that the coverage proportions of PCV13 (46%) and PPSV23 (66%) for isolates from all IPD cases (*n* = 291) were >10% lower than those detected for all IPD cases between April 2010 and March 2013. Collectively, these findings suggest the effects of immunization with PCV7 among children may have had indirect effects on adult IPD cases.

Serotypes 3, 19A, and 22F were the most common isolates among adult patients with IPD in our study. According to previous data, serotype 19A was frequently isolated from pediatric IPD cases in Japan, whereas serotypes 3 and 22F were rarely isolated from these cases between 2011 and 2013 [8]. After the introduction of PCV13 for children, serotype 19A (included in PCV13) appeared to decrease among adult patients with IPD overseas [15], and therefore we need to observe carefully any changes in serotype 19A isolation among adult IPD cases caused by the indirect effect of PCV13 among children by continuing the surveillance. Furthermore, we wonder whether the incidence of IPD caused by serotype 3 decreases after the introduction of PCV13 among children. Although a 44% reduction of IPD incidence caused by serotype 3 was reported in the UK among individuals who were aged 65 years or older after the introduction of PCV13 for children, a herd immunity effect against serotype 3 is unclear worldwide [15, 16]. In addition, the immunogenicity of PCV13 against serotype 3 remains controversial in children and was reported to be lower than that of PCV13 serotypes other than serotype 3 in the elderly [17–20].

The proportion of vaccine types was lower in immunocompromised patients than those in each age group of nonimmunocompromised patients in our study. This finding is consistent with those reported by Luján et al. [21]. Those authors reported that serotypes not included in PCV13/PPSV23 were isolated more frequently in patients with cardiac and respiratory comorbidities and in certain subgroups of immunocompromised patients, such as HIV-infected individuals and those with hematologic cancer. This finding suggests that monitoring the emergence of non-vaccine serotypes is crucial, especially in patients with specific underlying conditions.

This study had several limitations. The proportion of cases available for both case-report data and isolate data was 51% (291/567) among cases reported to national surveillance sites from the ten selected prefectures in this study, and reporting of some variables was incomplete, such as preceding influenza was not available for 34% of IPD patients; therefore, it may not represent the population of interest. The number of registered cases in the enhanced surveillance data increased annually (101 cases from April 2013 to March 2014 vs 202 cases from April 2014 to March 2015). Laboratory method or the procedure of specimen collection was unchanged during this period. The annual increase of registered cases might be explained by the improved flow of collecting isolates and case reporting forms from hospitals to NIID through local public health institutes during the study period, and heightened awareness of the IPD surveillance. This explanation is supported by the finding that the number of reported cases from the 10 selected prefectures to NESID increased during the study period (224 cases from April 2013 to March 2014 vs 343 cases from April 2014 to March 2015). The patients whose culture results were positive for *S. pneumoniae* in the normally sterile sites other than blood or CSF were not included because of the notification criteria of IPD in NESID; thus our results may be an underestimation.

## Conclusion

This study reported the characteristics of adult IPD and the serotype distribution of causative pathogens in Japan from April 2013 to March 2015. A lower proportion of PCV7-type IPD in our study period after PCV7 introduction in children compared to that in previous studies by others before PCV7 introduction suggests there may have been indirect effects of the infant immunization program on adult disease. The enhancement and continuation of the nationwide surveillance of IPD in adults are essential for evaluating IPD incidence and serotype distribution in adults, as well as the effect of herd immunity through the vaccination of children with PCV13.

## Additional files

Additional file 1: Distribution of the 10 prefectures participating in the enhanced surveillance. They are marked with numbers on a Japanese map: 1: Hokkaido; 2: Miyagi; 3: Yamagata; 4: Niigata; 5: Mie; 6: Nara; 7: Kochi; 8: Fukuoka; 9: Kagoshima; and 10: Okinawa prefecture. (PPTX 156 kb)
Additional file 2: Distribution of the serotypes of causative pneumococcal isolates from patients with invasive pneumococcal diseases by age and immunocompromised status in four groups (*n* = 281); Group 1 (15-39y, nonimmunocompromised) *n* = 10 (A), Group 2 (40-64y, nonimmunocompromised) *n* = 48 (B), Group 3 (> = 65y, nonimmunocompromised) *n* = 116 (C), Group 4 (> = 15y immunocompromised) *n* = 107 (D). (PPTX 74 kb)

## Abbreviations

BMI: Body mass index
CSF: Cerebrospinal fluid
IPD: Invasive pneumococcal disease
NESID: National epidemiological surveillance of infectious diseases
PCV: Pneumococcal conjugate vaccine
PPSV: Pneumococcal polysaccharide vaccine
