# Supplementary figures and images for: Invasive pneumococcal disease among adults in Japan, April 2013 to March 2015: disease characteristics and serotype distribution
Source: BMC Infect Dis. 2017 Jan 3;17:2. doi: 10.1186/s12879-016-2113-y (PMC5209803; doi:10.1186/s12879-016-2113-y)

## Slide 1
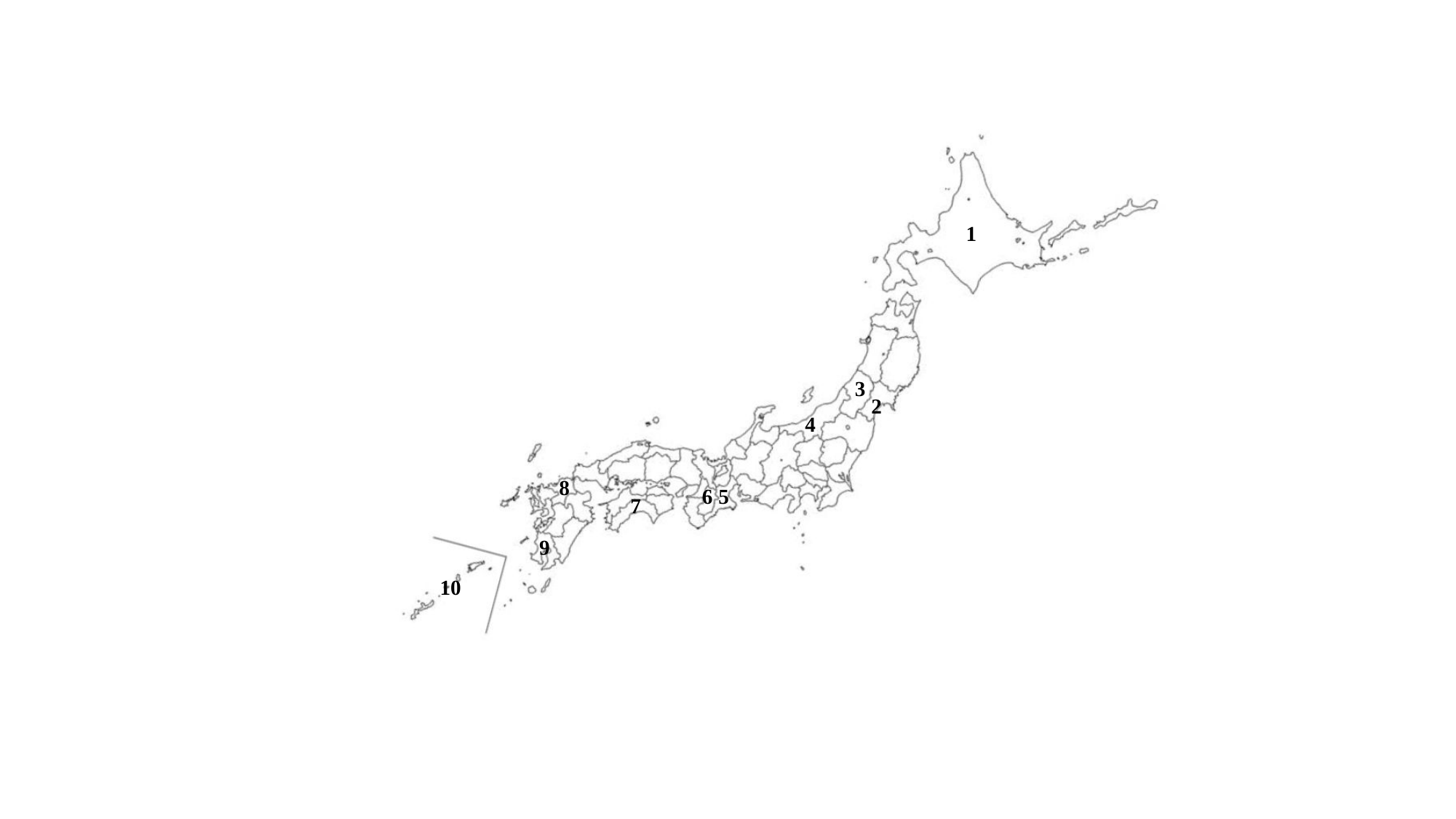

1
2
4
8
5
7
9
10
3
6

Supplement: Additional file 1: — Distribution of the 10 prefectures participating in the enhanced surveillance. They are marked with numbers on a Japanese map: 1: Hokkaido; 2: Miyagi; 3: Yamagata; 4: Niigata; 5: Mie; 6: Nara; 7: Kochi; 8: Fukuoka; 9: Kagoshima; and 10: Okinawa prefecture. (PPTX 156 kb) [file 12879_2016_2113_MOESM1_ESM.pptx]
