# Supplementary material for: Invasive pneumococcal disease among adults in Japan, April 2013 to March 2015: disease characteristics and serotype distribution
Source: BMC Infect Dis. 2017 Jan 3;17:2. doi: 10.1186/s12879-016-2113-y (PMC5209803; doi:10.1186/s12879-016-2113-y)
Supplement: Additional file 2: — Distribution of the serotypes of causative pneumococcal isolates from patients with invasive pneumococcal diseases by age and immunocompromised status in four groups (n = 281); Group 1 (15-39y, nonimmunocompromised) n = 10 (A), Group 2 (40-64y, nonimmunocompromised) n = 48 (B), Group 3 (> = 65y, nonimmunocompromised) n = 116 (C), Group 4 (> = 15y immunocompromised) n = 107 (D). (PPTX 74 kb) [file 12879_2016_2113_MOESM2_ESM.pptx]

## Slide 1
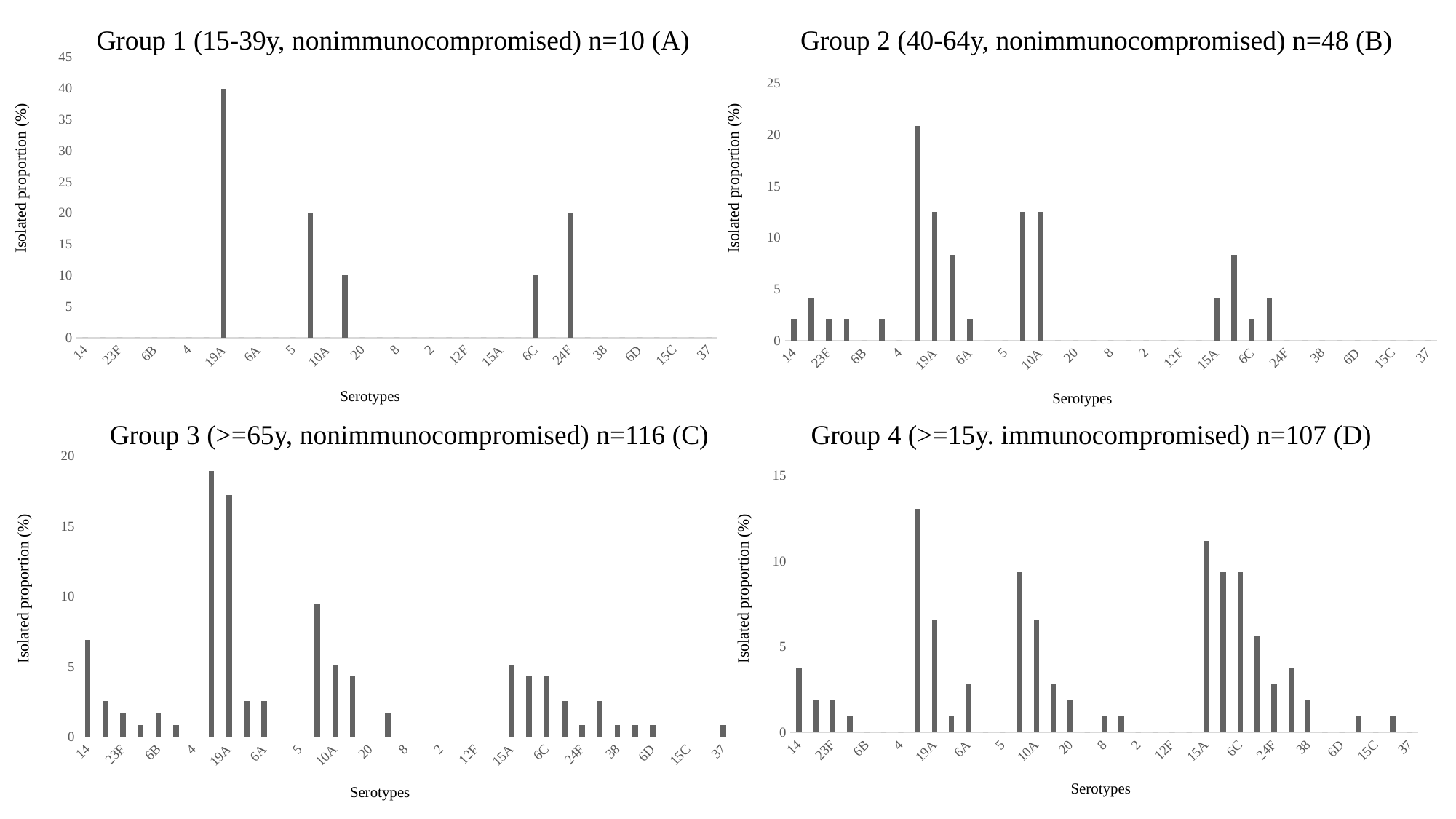

Group 2 (40-64y, nonimmunocompromised) n=48 (B)
Group 1 (15-39y, nonimmunocompromised) n=10 (A)
### Chart
| Category | |
|---|---|
| 14 | 0.0 |
| 19F | 0.0 |
| 23F | 0.0 |
| 9V | 0.0 |
| 6B | 0.0 |
| 18C | 0.0 |
| 4 | 0.0 |
| 3 | 0.0 |
| 19A | 40.0 |
| 7F | 0.0 |
| 6A | 0.0 |
| 1 | 0.0 |
| 5 | 0.0 |
| 22F | 20.0 |
| 10A | 0.0 |
| 11A/E | 10.0 |
| 20 | 0.0 |
| 33F | 0.0 |
| 8 | 0.0 |
| 15B | 0.0 |
| 2 | 0.0 |
| 9N | 0.0 |
| 12F | 0.0 |
| 17F | 0.0 |
| 15A | 0.0 |
| 23A | 0.0 |
| 6C | 10.0 |
| 35B | 0.0 |
| 24F | 20.0 |
| 34 | 0.0 |
| 38 | 0.0 |
| 7C | 0.0 |
| 6D | 0.0 |
| 13 | 0.0 |
| 15C | 0.0 |
| 18B | 0.0 |
| 37 | 0.0 |
### Chart
| Category | |
|---|---|
| 14 | 2.083333333333334 |
| 19F | 4.166666666666667 |
| 23F | 2.083333333333334 |
| 9V | 2.083333333333334 |
| 6B | 0.0 |
| 18C | 2.083333333333334 |
| 4 | 0.0 |
| 3 | 20.83333333333328 |
| 19A | 12.5 |
| 7F | 8.333333333333336 |
| 6A | 2.083333333333334 |
| 1 | 0.0 |
| 5 | 0.0 |
| 22F | 12.5 |
| 10A | 12.5 |
| 11A/E | 0.0 |
| 20 | 0.0 |
| 33F | 0.0 |
| 8 | 0.0 |
| 15B | 0.0 |
| 2 | 0.0 |
| 9N | 0.0 |
| 12F | 0.0 |
| 17F | 0.0 |
| 15A | 4.166666666666667 |
| 23A | 8.333333333333336 |
| 6C | 2.083333333333334 |
| 35B | 4.166666666666667 |
| 24F | 0.0 |
| 34 | 0.0 |
| 38 | 0.0 |
| 7C | 0.0 |
| 6D | 0.0 |
| 13 | 0.0 |
| 15C | 0.0 |
| 18B | 0.0 |
| 37 | 0.0 |Isolated proportion (%)
Isolated proportion (%)
Serotypes
Serotypes
Group 4 (>=15y. immunocompromised) n=107 (D)
Group 3 (>=65y, nonimmunocompromised) n=116 (C)
### Chart
| Category | |
|---|---|
| 14 | 6.896551724137931 |
| 19F | 2.586206896551724 |
| 23F | 1.724137931034483 |
| 9V | 0.862068965517241 |
| 6B | 1.724137931034483 |
| 18C | 0.862068965517241 |
| 4 | 0.0 |
| 3 | 18.96551724137923 |
| 19A | 17.24137931034483 |
| 7F | 2.586206896551724 |
| 6A | 2.586206896551724 |
| 1 | 0.0 |
| 5 | 0.0 |
| 22F | 9.482758620689655 |
| 10A | 5.172413793103448 |
| 11A/E | 4.310344827586195 |
| 20 | 0.0 |
| 33F | 1.724137931034483 |
| 8 | 0.0 |
| 15B | 0.0 |
| 2 | 0.0 |
| 9N | 0.0 |
| 12F | 0.0 |
| 17F | 0.0 |
| 15A | 5.172413793103448 |
| 23A | 4.310344827586195 |
| 6C | 4.310344827586195 |
| 35B | 2.586206896551724 |
| 24F | 0.862068965517241 |
| 34 | 2.586206896551724 |
| 38 | 0.862068965517241 |
| 7C | 0.862068965517241 |
| 6D | 0.862068965517241 |
| 13 | 0.0 |
| 15C | 0.0 |
| 18B | 0.0 |
| 37 | 0.862068965517241 |
### Chart
| Category | |
|---|---|
| 14 | 3.738317757009347 |
| 19F | 1.869158878504673 |
| 23F | 1.869158878504673 |
| 9V | 0.934579439252336 |
| 6B | 0.0 |
| 18C | 0.0 |
| 4 | 0.0 |
| 3 | 13.08411214953271 |
| 19A | 6.542056074766355 |
| 7F | 0.934579439252336 |
| 6A | 2.80373831775701 |
| 1 | 0.0 |
| 5 | 0.0 |
| 22F | 9.34579439252337 |
| 10A | 6.542056074766355 |
| 11A/E | 2.80373831775701 |
| 20 | 1.869158878504673 |
| 33F | 0.0 |
| 8 | 0.934579439252336 |
| 15B | 0.934579439252336 |
| 2 | 0.0 |
| 9N | 0.0 |
| 12F | 0.0 |
| 17F | 0.0 |
| 15A | 11.21495327102804 |
| 23A | 9.34579439252337 |
| 6C | 9.34579439252337 |
| 35B | 5.607476635514017 |
| 24F | 2.80373831775701 |
| 34 | 3.738317757009347 |
| 38 | 1.869158878504673 |
| 7C | 0.0 |
| 6D | 0.0 |
| 13 | 0.934579439252336 |
| 15C | 0.0 |
| 18B | 0.934579439252336 |
| 37 | 0.0 |Isolated proportion (%)
Isolated proportion (%)
Serotypes
Serotypes
